# Structural basis for allosteric agonism of human α7 nicotinic acetylcholine receptors

**DOI:** 10.1038/s41421-025-00788-y

**Published:** 2025-04-08

**Authors:** Sanling Liu, Yining Zheng, Haopeng Chen, Xin Li, Qipeng Yan, Wenjun Mu, Yaning Fu, Huan Chen, Hongwei Hou, Lei Liu, Changlin Tian

**Affiliations:** 1 Division of Life Sciences and Medicine, Joint Center for Biological Analytical Chemistry, Anhui Engineering Laboratory of Peptide Drug, University of Science and Technology of China, Hefei, Anhui China; 2 Beijing Life Science Academy, Beijing, China; 3 Department of Chemistry, Tsinghua University, Beijing, China; 4 School of Chemistry and Chemical Engineering, Zhangjiang Institute for Advanced Study, Shanghai Jiao Tong University, Shanghai, China; 5 School of Biomedical Engineering, Suzhou Institute for Advanced Research, University of Science and Technology of China, Suzhou, Jiangsu China

**Keywords:** Cryoelectron microscopy, Ion channel signalling

## Abstract

The α7 nicotinic acetylcholine receptor (nAChR), a pentameric ligand-gated ion channel, plays important roles in cognition, neuroprotection, and anti-inflammation. As a potential drug target, α7 nAChR has different binding sites for different ligands, particularly agonists and positive allosteric modulators (PAMs). Ago-PAMs can both directly activate and allosterically modulate α7 nAChR. However, the mechanism underlying α7 nAChR modulation by ago-PAM has yet to be fully elucidated. Here, we present cryo-EM structures of α7 nAChR in complex with the ago-PAM GAT107 and Ca^2+^ in the open and desensitized states, respectively. Our results from both structural comparisons and functional assays suggest an allosteric mechanism underlying GAT107 modulation and calcium potentiation of α7 nAChR, involving local conformational changes in the ECD–TMD coupling region and a global structural rearrangement in the transmembrane domain. This work provides a new mechanism of α7 nAChR gating distinct from that of conventional agonist binding. These findings would aid in drug design and enrich our biophysical understanding of pentameric ligand-gated ion channels.

## Introduction

The nicotinic acetylcholine receptor (nAChR) belongs to the pentameric ligand-gated ion channel (pLGIC) superfamily and has a variety of subtypes^1^. Homopentameric α7 nAChR is one of the most abundant nAChRs in the nervous system and is found in neurons as well as non-neuronal cells, including microglia and astrocytes^2–4^. α7 nAChR is not only highly permeable to calcium but also potentiated by external calcium^5^. α7 nAChR is involved in neuroprotection, cognitive function, and reward pathways^6–8^. Dysregulation of the receptor has been linked to a spectrum of neurological and psychiatric disorders, such as Alzheimer’s disease^9,10^, Parkinson’s disease^11,12^, and schizophrenia^13,14^. In addition, α7 nAChR is also found in organs and tissues of the immune system^15^ and acts as a fundamental component of the cholinergic anti-inflammatory pathway^16–20^. Many studies have shown that α7 nAChR activation can effectively attenuate pro-inflammatory cytokine production in macrophages and microglia^21,22^. Thus, α7 nAChR has emerged as a potential therapeutic target for neurologic and inflammatory disorders^23^.

The most straightforward strategy in α7 nAChR-targeted drug development involves identifying selective α7 nAChR agonists. However, these agonists predominantly lead to non-conducting, desensitized receptor states, which diminishes the efficacy of the drugs because of a thermodynamic shift towards this end state^24^. Moreover, the development of the compound encenicline (EVP-6124), a quinuclidine derivative with high affinity for α7 nAChRs, was halted after phase III trials because severe gastrointestinal side effects occurred in elderly individuals^25–28^. Additionally, chronic agonist exposure results in compensatory changes in α7 nAChR receptor regulation^29^. Therefore, positive allosteric modulators (PAMs), which are also ligands of α7 nAChRs, are gaining attention for their ability to selectively increase receptor activity^30,31^. Most PAMs, such as type I and type II PAMs, are unable to activate receptors on their own^32–34^. However, ago-PAMs (also called allosteric agonists), such as GAT107, are unique. Ago-PAMs are capable of directly activating α7 receptors and inducing a sustained potentiation of responses evoked by orthosteric agonists^35,36^. This ability to both directly activate and allosterically modulate α7 nAChR is significant and obviously different from that of agonists or other PAMs of α7 nAChRs^37–39^.

In recent years, the structure and gating mechanism of α7 nAChR coupled with agonist or agonist/PAM binding have been reported^40,41^. In contrast, the binding site for ago-PAM has been a subject of controversy^37,42^ until recently, when Burke et al. reported cryo-EM structures of α7 nAChR complexed with the ago-PAM GAT107 in the absence or presence of an agonist^43^. However, the mechanism underlying the modulation of α7 nAChR upon ago-PAM binding remains to be explored further. Here, we report the cryo-EM structures of the human α7 nAChR in complex with GAT107 and Ca^2+^ in the open and desensitized states. By combining these two structures with previously reported structures^41,43^, the structural mechanism behind GAT modulation and calcium potentiation of α7 nAChR is elucidated.

## Results

### Structures of α7 nAChR in complex with GAT107 and Ca^2+^

**Fig. 1:**
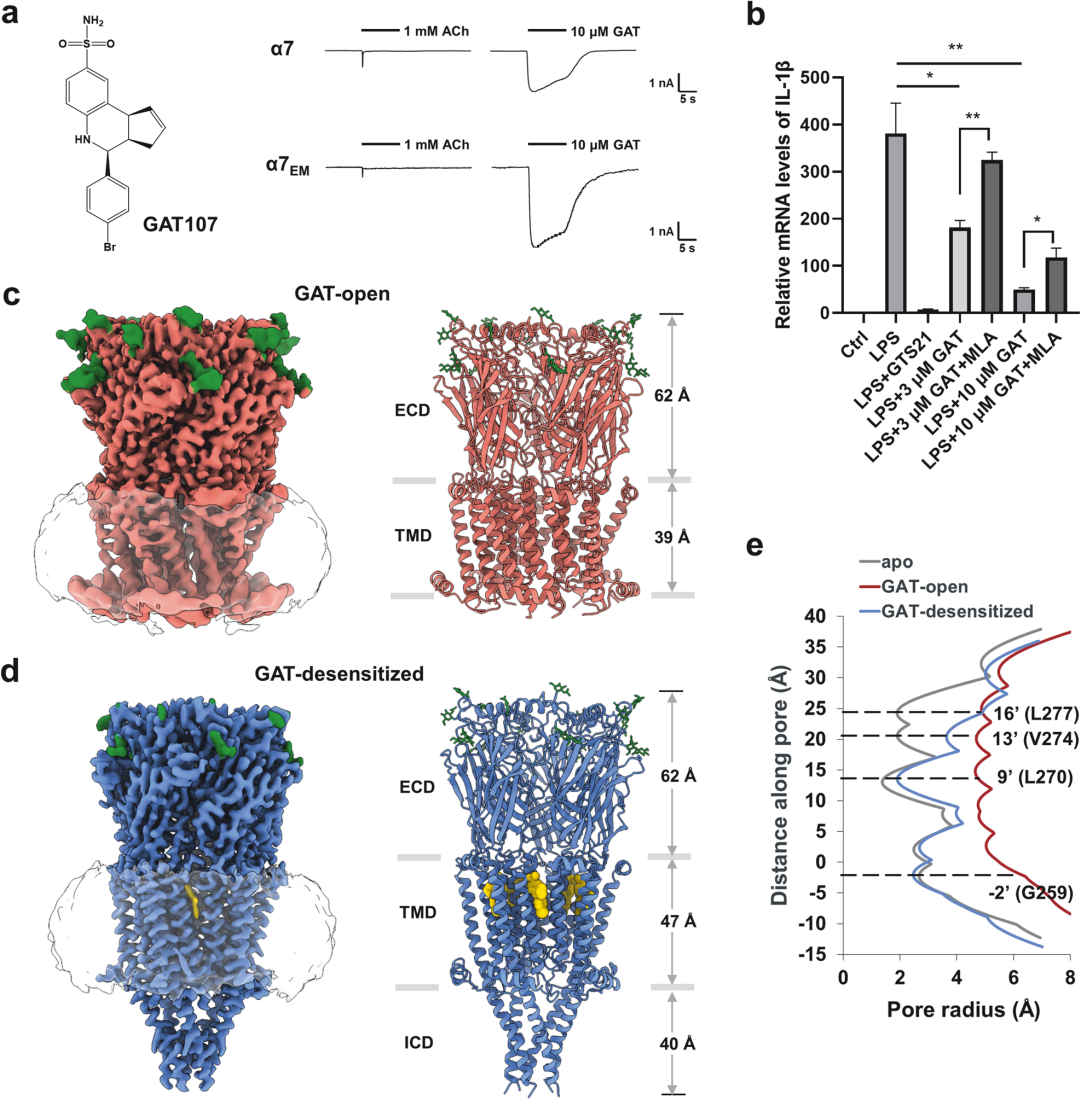
Cryo-EM structures of the human α7 nAChR in complex with GAT107 and Ca^2+^. **a** Chemical structure of GAT is shown. Representative responses of α7 or α7_EM_ nAChR are shown for the application of ACh or GAT in bath solutions containing 2 mM Ca^2+^ with a 10-s duration. **b** Effect of GAT on the release of inflammatory molecule IL-1β in LPS-stimulated microglial BV2 cells. The concentrations of LPS, GTS21, and MLA used in the experiment are 1 μg/mL, 100 μM, and 10 μM, respectively. Data are shown as means ± SEM (*n* ≥ 3). *P*-values were calculated using an unpaired two-tailed Student’s *t*-test. *P < 0.05, **P < 0.01. **c** Cryo-EM maps and ribbon representation of the complex in the open state. The Asn-linked carbohydrates and associated residues (Asn46, Asn90, and Asn133) are shown as sticks (green). **d** Cryo-EM maps and ribbon representation of the complex in a desensitized state. The Asn-linked carbohydrates and associated residues (Asn46, Asn90, and Asn133) are shown as sticks (green). GAT molecules are shown as spheres (yellow). **e** Plots of pore radius for α7 receptors along the pore axis. The α-carbon position of 0’ (Lys261) is set to zero. Channel pore radius was calculated using the HOLE program.

GAT107 (abbreviated as GAT) is the active isomer of 4BP-TQS and exhibits high affinity and selectivity towards α7 nAChR as an allosteric agonist or ago-PAM^44–46^. Whole-cell currents evoked by GAT were recorded in HEK293T cells expressing human α7 nAChR or α7_EM_ nAChR (the construct used for structural determination, see Materials and Methods), with Ca^2+^ included in the bath solution. As expected^35^, GAT elicited substantial activation of α7 receptors. The currents evoked by GAT rapidly reached a peak, significantly larger than that of the control response induced by acetylcholine (ACh) (Fig. 1a). Upon prolonged exposure to GAT, the currents decayed slowly for both α7 and α7_EM_ nAChR. These results indicate that α7 nAChRs undergo slow desensitization in response to prolonged GAT application. Additionally, we tested the effects of GAT on lipopolysaccharide (LPS)-stimulated microglial BV2 cells. GAT significantly reduced the production of pro-inflammatory molecules such as IL-1β in a dose-dependent manner, and this effect was reversed by methyllycaconitine (MLA), which is a selective antagonist of α7 nAChR (Fig. 1b). These findings reveal that GAT has a prospective role in alleviating neuroinflammatory responses.

To gain insight into the structural basis underlying the unique properties of ago-PAM and Ca^2+^ at α7 nAChRs, we determined the cryo-EM structures of α7 nAChR in the presence of both GAT and Ca^2+^. The α7 nAChR protein was prepared as previously reported^41^. Following the collection and processing of cryo-EM images, we successfully obtained two distinct 3D reconstructions with global resolutions of 2.89 Å and 2.93 Å, respectively (Fig. 1; Supplementary Figs. S1, S2 and Table S1). We measured the pore diameter in these structures (Fig. 1e) and compared them with those from previous studies^40,41^. The results indicated that these two structures represented an open state and a desensitized state, respectively. The local resolution of the open-state map varied greatly across the receptor and was highest at the extracellular domain (ECD) (Supplementary Fig. S1d). In the ECDs of these two structures, three *N*-linked glycosylation sites (Asn46, Asn90, and Asn133) were present in each subunit (Fig. 1c, d). The structure of the intracellular domain in the open state was not resolved because of its ambiguous density. In the desensitized-state structure, all the domains in the receptor together with the molecule GAT were well resolved. The thickness of the transmembrane domain (TMD) along the central channel axis in the open state (39 Å) was markedly shorter than that in the desensitized state (47 Å) or the apo form (48 Å), indicating a significant rearrangement of the transmembrane helices during the activation and desensitization of α7 nAChR by GAT and Ca^2+^.

### Structures of the ECDs and ECD–TMD coupling regions

**Fig. 2:**
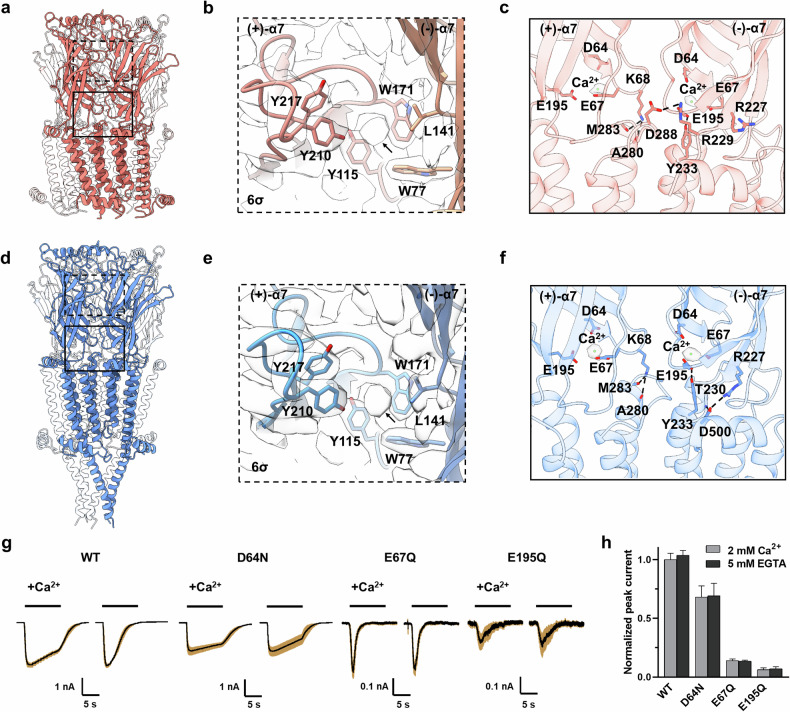
Structures of the ECDs and ECD–TMD coupling regions in the GAT-open and desensitized states. **a** Structure of the GAT-open state viewed from the membrane plane. For clarity, all but two of the subunits are mostly transparent. **b** Zoomed-in view of the ECDs as in **a**, viewed from the extracellular side. Corresponding densities are contoured at 6σ (standard deviations). Residues involved in neurotransmitter binding are shown in sticks. The extra density in the orthosteric binding pocket is indicated by the arrow. **c** The structural details of the ECD–TMD interface between two adjacent subunits. Electrostatic interactions are represented as dashed lines. Corresponding densities for Ca^2+^ are contoured at 5σ. **d** Structure of GAT-desensitized state viewed from the membrane plane. **e** Zoomed-in view of the ECDs as in **d**, viewed from the extracellular side. Corresponding densities are contoured at 6σ. Residues involved in neurotransmitter binding are shown in sticks. The extra density in the orthosteric binding pocket is indicated by the arrow. **f** The structural details of the ECD–TMD interface between two adjacent subunits. Electrostatic interactions are represented as dashed lines. Corresponding densities for Ca^2+^ are contoured at 5σ. **g** Responses of α7 nAChR and its mutants (D64N, E67Q, and E195Q) expressed in HEK293T cells to GAT in the presence or absence of Ca^2+^ are shown. The averaged responses (black solid lines) along with the standard error of the mean (SEM, brown shaded area) are illustrated (*n* ≥ 3). **h** The statistical analyses comparing the peak currents triggered by GAT in the presence and absence of Ca^2+^ are presented.

The ECDs of both the GAT-open and desensitized states show similar conformations to those of apo-α7 nAChR, which is attributable to the empty orthosteric pocket (Fig. 2a, d). Close inspection reveals slight closure of loop C in the GAT-open and -desensitized states compared with apo-α7 (Supplementary Fig. S3a, b). Intriguingly, we observed a clear microspheric density in the neurotransmitter-binding site, surrounded by Y115 in loop A, W171 in loop B, Y210 and Y271 in loop C, and W77 in loop D, in both the open and desensitized states (Fig. 2b, e). This density is apparently not compatible to the GAT molecule. Initially, we speculated that this density might represent a calcium ion. However, a relatively weaker density was also discernible at the corresponding position in the apo-α7 nAChR structure^41^ in the absence of added calcium ions (Supplementary Fig. S3c). A small spherical density was also observed at a similar site in the α7_ECD_/nanobody structure, which has been proposed to be either a water molecule or a cation^47^. Taken together, the identity of this density remains unidentified.

In the ECD–TMD coupling region, a well-defined density for a Ca^2+^ was found in both the GAT-open and desensitized states (Fig. 2c, f), which is consistent with the structure of the α7/epi/PNU complex^40^. The putative Ca^2+^ is adjacent to the side chains of D64, E67 and E195. Previous electrophysiological studies showed that mutations at E67 or E195 affect the calcium potentiation of ACh-induced currents in the α7-5HT_3_ chimera^48^. To validate the influence of Ca^2+^ on GAT-induced activation, we performed electrophysiology experiments on both the α7 nAChR and its mutants D64N, E67Q, and E195Q. As shown in Fig. 2g, h, although the peak currents induced by GAT remained indistinguishable, the rates of desensitization revealed notable disparities for α7 nAChR in the presence and absence of Ca^2+^. In contrast to the responses to GAT with Ca^2+^, prolonged exposure to GAT in a Ca^2+^-free environment resulted in a significantly faster desensitization rate. For the D64N mutant, the response to GAT in the absence of Ca^2+^ exhibited a relatively slower desensitization rate than the wild-type (WT) receptor, with no noticeable differences between conditions with and without Ca^2+^. For the E67Q and E195Q mutants, the currents evoked by GAT were substantially lower than those of the WT receptor, and the Ca^2+^-potentiating effect was abolished. Taken together, due to the key locations of residues D64, E67, and E195 in the ECD–TMD coupling region, mutations at these sites affect the current elicited by GAT. Moreover, these mutants also exhibit a loss of the potentiating effect of Ca^2+^ on GAT activation. These results further corroborate that the density adjacent to D64, E67, and E195 is indeed attributed to a calcium ion.

An intersubunit interaction between K68 in the primary (+) subunit and E195 in the complementary (–) subunit, which is pivotal in the resting state, is disrupted in the GAT-open structure (Fig. 2c). The side chain of K68 pivots along with its β1–β2 loop to engage in an intrasubunit interaction with the carbonyl oxygen of A280 and M283 at the extracellular terminus of M2. Moreover, (+)-D288 in the M2–M3 loop electrostatically interacts with (–)-R229 in the pre-M1 linker. The side chain of E195 establishes contacts with Y233 in M1 of the same subunit, suggesting a fortified coupling between the ECD and TMD. These intrasubunit interactions contributed by K68–A280, K68–M283, and E195–Y233 still exist in the GAT-desensitized state (Fig. 2f). In addition, D500 in the C-terminal helix parallel to the membrane panel interacts with R227 and the nitrogen of T230 in the desensitized state. In contrast, the C-terminal helix deviates away from the pre-M1 linker, leading to a relatively loose helix bundle in the open state.

### GAT binds to the TMD

**Fig. 3:**
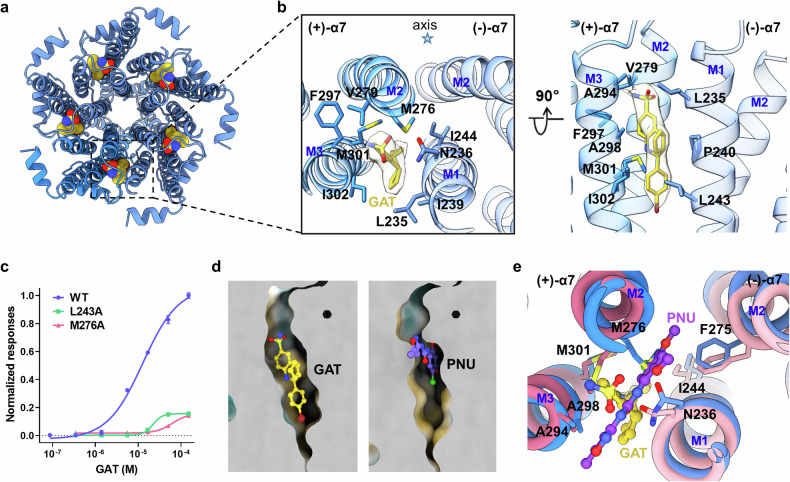
GAT binds to the TMD. **a** Atomic model overview of the binding sites for GAT in the GAT-desensitized structure. GAT molecules are shown as spheres. **b** Molecular details of the GAT-binding interface viewed from the extracellular space (left) and parallel to the membrane plane (right). GAT is shown as sticks with corresponding density. The star symbol represents the pore axis. **c** The effects of mutations in α7 nAChR on the response to GAT were evaluated using calcium flux measurements. **d** The allosteric binding pockets for GAT and PNU. **e** Comparison of the binding sites for GAT (blue and yellow) and PNU (pink and magenta, PDB: 8V82), viewed from the extracellular space.

In the desensitized-state structure, five GAT molecules are observed within the pentameric TMD (Fig. 3a), each located in a hydrophobic pocket consisting of residues in helices M2 and M3 of the primary subunit and in the M1 helix of the complementary subunit (Fig. 3b). Specifically, the brominated aromatic group of GAT inserts vertically in the pocket and is in close proximity to M301 and I302 in (+)-M3 and L243 and I244 in (–)-M1. The polycyclic aromatic group in the middle position of GAT interacts with M276 in (+)-M2, F297, and A298 in (+)-M3, and I239 and P240 in (–)-M1. Moreover, the sulfonamide group of GAT points upwards and makes electrostatic interactions with N236 in (–)-M1. The GAT sulfonamide group is also close to V279 in (+)-M2, A294 in (+)-M3, and L235 in (–)-M1. The calcium flux measurement data revealed that the efficiency and response to GAT of the L243A and M276A mutants were dramatically lower than those of the WT α7 nAChR (Fig. 3c), which is consistent with the observed structure.

The binding pocket for GAT is in a cavity comparable to that for the type II PAM PNU-120596 (abbreviated as PNU) identified in earlier studies^40,41^. GAT and PNU occupy partially overlapping sites at the intersubunit interfaces within the TMD. However, the orientations of these two molecules are very different. GAT inserts in the pocket perpendicularly to the membrane, whereas PNU displays horizontal binding in the TMD (Fig. 3d, e). Owing to the distinct difference in binding mode, GAT has more interactions with helices M1 and M3 and fewer interactions with helix M2 than PNU does (Supplementary Fig. S4a). This configuration causes GAT to be far from the channel pore. Notably, the vertical depth along the channel pore of GAT is almost twice as deep as that of PNU (Supplementary Fig. S4b). GAT occupies a position spanning from the middle to the top of the transmembrane helix. Although both GAT and PNU are in close proximity to M276 in (+)-M2, the orientation of the side chain of M276 is quite distinct. The side chain of M276 in the GAT-desensitized structure is consistent with that in apo-α7, while it exhibits a rotational shift in the PNU-bound complex. Analysis of M276 mutation to alanine or leucine reveals a significant impact on GAT and PNU response (Supplementary Fig. S4c). GAT binding to the M276A mutant led to a more significant reduction in receptor activity than GAT binding to the M276L mutant when compared with the WT receptor. In contrast, M276L had a greater impact on PNU binding than M276A. This finding reveals that the flexibility of the M276 side chain plays an essential role in PNU binding, which was also reported by Burke et al.^43^ Instead, the direct interaction between M276 and GAT plays an important role in the activity of this molecule.

Although GAT was unambiguously assigned in the desensitized-state structure, its density was not discernible in the open-state map. This phenomenon is reminiscent of what was observed with PNU in the epi/PNU-bound α7 open structure^40^. The results of molecule docking and molecular dynamics (MD) simulations revealed that the RMSD values of GAT molecules increased gradually in the docked open-state complex throughout the simulations (Supplementary Fig. S5). In the final frame, all five GAT molecules displayed various conformations distinct from those in the initial frame. In contrast, in the desensitized state, the GAT molecules in the final frame occupied the allosteric pocket, maintaining conformations similar to those observed in the cryo-EM structure. This finding indicates that GAT binds stably in the desensitized state, but exhibits reduced stability in the open state. It is hypothesized that the highly dynamic binding mode of the GAT molecules in the open state precludes the visualization of these small molecules.

### Structures of the TMDs

Compared with the α7/epi/PNU complex^40^, which is the only open-state structure of the α7 receptor reported to date, the GAT-open state here exhibits an overall similar channel pore but with some differences in detail (Supplementary Fig. S6). Helices M2 and M3 and the upper half of M1 in the GAT-open structure display anticlockwise translocation relative to the epi/PNU-open structure, whereas there are few differences in helix M4. These structural differences could be attributed to several factors, such as distinct membrane environments (lipid or detergent) and different effects of PNU and GAT on the receptor.

**Fig. 4:**
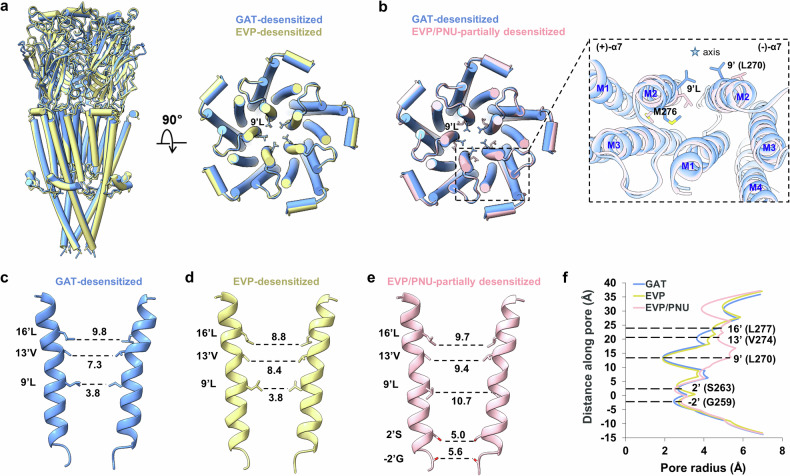
Structural comparisons of the TMDs in the GAT-desensitized state with that in the EVP- or EVP/PNU-bound state. **a** Superimposed GAT-desensitized structure (blue) and α7/EVP structure (yellow, PDB: 7EKP), viewed from the membrane plane (left) and the extracellular side (right), respectively. **b** Superimposed TMDs of GAT-desensitized structure (blue) and α7/EVP/PNU structure (pink, PDB: 7EKT), viewed from the extracellular side. **c**–**e** Two opposite M2 helices and pore diameters (Å) are shown in GAT-desensitized (**c**), α7/EVP (**d**), or α7/EVP/PNU (**e**) structure. **f** Plots of pore radius for α7 receptors along the pore axis. The α-carbon position of 0’ (Lys261) is set to zero. Channel pore radius was calculated using the HOLE program.

Interestingly, although there are notable differences in the ECDs between the GAT-desensitized state and the agonist EVP-bound α7 structure, the extensive electrostatic interactions in the ECD–TMD coupling region are almost the same in these two structures (Fig. 4a; Supplementary Fig. S7). The TMD of the GAT-desensitized structure closely resembles that of the EVP-desensitized state, characterized by tight hydrophobic constraints at the 9’ (L270) position and expansion at the 16’ (L277) site within the channel pore (Fig. 4c, d, f). Further structural comparisons between the TMDs of the GAT-desensitized state and EVP/PNU-bound α7 highlight the significant rotation of M276, which in turn expands the channel pore proximal to 9’L in the α7/EVP/PNU structure (Fig. 4b, e). This observation suggests that the 9’L rotation is instrumental in the modulatory action of PNU. These findings imply that the modulatory mechanism of GAT diverges from that of PNU.

### TMD rearrangements upon GAT107 and Ca^2+^ binding

**Fig. 5:**
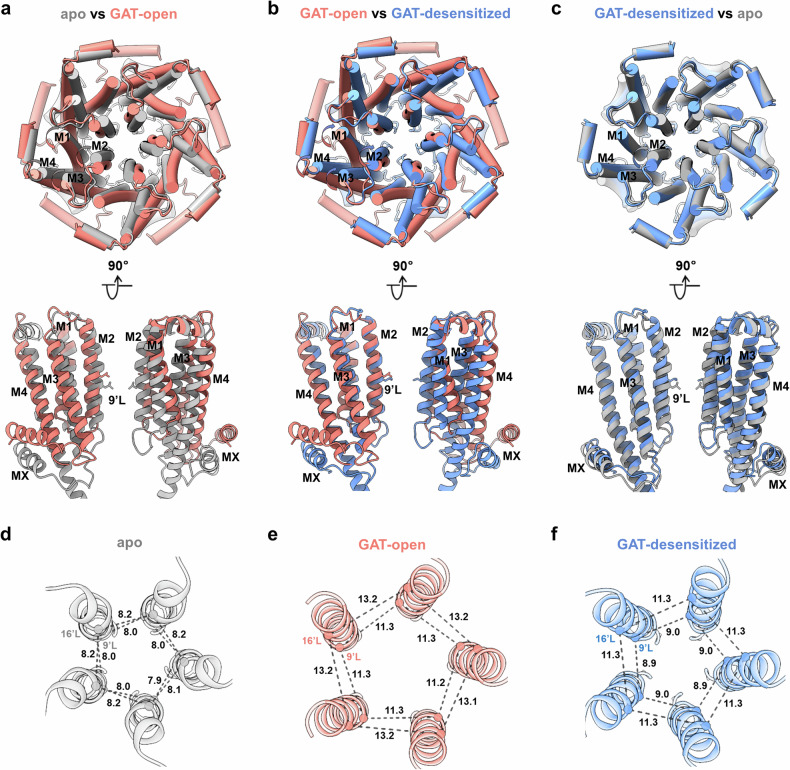
TMD rearrangements upon GAT and Ca^2+^ binding. **a** Superimposition of the TMDs from the apo-α7 (gray, PDB: 7EKI) and GAT-open-state (orange) structures. **b** Superimposition of the TMDs from the GAT-open-state (orange) and GAT-desensitized-state (blue) structures. **c** Superimposition of the TMDs from GAT-desensitized-state (blue) and the apo-α7 (gray) structures. **d** A view of M2 helices from the extracellular space in the apo-α7 structure. The corresponding distances between adjacent α-carbons of 9’ (L270) and 16’ (L277) are given in Å. **e**, **f** A view of M2 helices from the extracellular space in the GAT-open-state (**e**) or GAT-desensitized-state (**f**) structure.

To elucidate the conformation transition of α7 nAChR in the presence of GAT and Ca^2+^, we conducted a comparative analysis of the TMDs of the receptor between the GAT-open and desensitized states, along with the apo-α7 structure (Fig. 5).

The TMDs undergo remarkable rearrangements upon GAT and Ca^2+^ binding. During activation, helices M1, M2 and M3 undergo anticlockwise rotation, lateral outwards translation and a coordinated tilt. The helix MX ascends, and M4 tilts and shifts outwards, facilitating the movement of M1–M3 (Fig. 5a). The above conformational changes lead to a decrease of the thickness of the TMD and overall expansion throughout the channel pore (Fig. 1c–e). When the receptor changes from an open state to a desensitized state, the helices in the TMD undergo a clockwise translocation, and almost all of them revert to a resting-like conformation, except for helix M2 (Fig. 5b). This results in a typical desensitized channel pore of α7 nAChR, with the lower half resembling that of apo-α7, and the upper half more similar to that in the open channel (Fig. 1e). Once helix M2 undergoes clockwise rotation and descends, the upper half of the pore contracts, and the channel returns to the resting state (Fig. 5c).

## Discussion

We obtained two well-resolved structures in the cryo-EM dataset of α7 nAChR complexed with GAT and Ca^2+^. These two structures are considered to be in an open and desensitized state, respectively. Recently, Burke et al. reported cryo-EM structures of GAT-bound α7 nAChR in a resting-like state and epi/GAT-bound α7 nAChR in a “desensitized-intermediate” state^43^ when this manuscript was in preparation. The position of GAT and its interacting residues align quite well with the reported structures^44^ and the GAT-desensitized-state structure in this study. The striking differences between the GAT-resting-state and GAT-desensitized-state structures exist in the upper half of helix M2 (Supplementary Fig. S8a), resulting in two distinct channel pore conformations (Supplementary Fig. S8e). We also notice that the Ca^2+^ adjacent to D64, E67, and E195 in the ECD–TMD coupling region of the GAT-desensitized state are not present in the GAT-resting-state structure. In contrast, all the helices in the TMDs in α7/epi/GAT align well with those in the GAT-desensitized-state structure (Supplementary Fig. S8b). There are differences in the side chains of the pore-lining residues in the M2 helix between the α7/epi/GAT and GAT-desensitized structures (Supplementary Fig. S8d), especially at the 9’L site. Compared with the well-defined density corresponding to 9’L in the GAT-resting-state and GAT-desensitized-state maps, the density is relatively ambiguous in the epi/GAT map (Supplementary Fig. S8c). Therefore, the pore conformation of the GAT-desensitized-state structure in this study is apparently different from that of the GAT-resting or epi/GAT-desensitized-intermediate states. This difference indicates that the molecular mechanism by which α7 nAChR is modulated by GAT is affected by different factors, such as Ca^2+^ and agonists.

To our knowledge, most of the electrophysiological experiments on α7 nAChR activation by GAT reported previously were performed in a bath solution containing Ca^2+^ ^35–37^. Here we also evaluated the effect of GAT on receptor activation under calcium-free conditions. The whole-cell patch clamp recording data indicated that GAT induced a weakly decaying current in α7 nAChR when Ca^2+^ was present, in contrast to conditions where Ca^2+^ was absent (Fig. 2g). This Ca^2+^-dependent potentiation may account for our success in capturing the open-state structure of the receptor in this study. We also obtained a closed-pore structure with a plot of pore diameters closely resembling that of agonist-bound α7 nAChR in desensitized states (Fig. 4f)^40,41^. Based on this similarity, we have assigned this structure as representing a desensitized state.

**Fig. 6:**
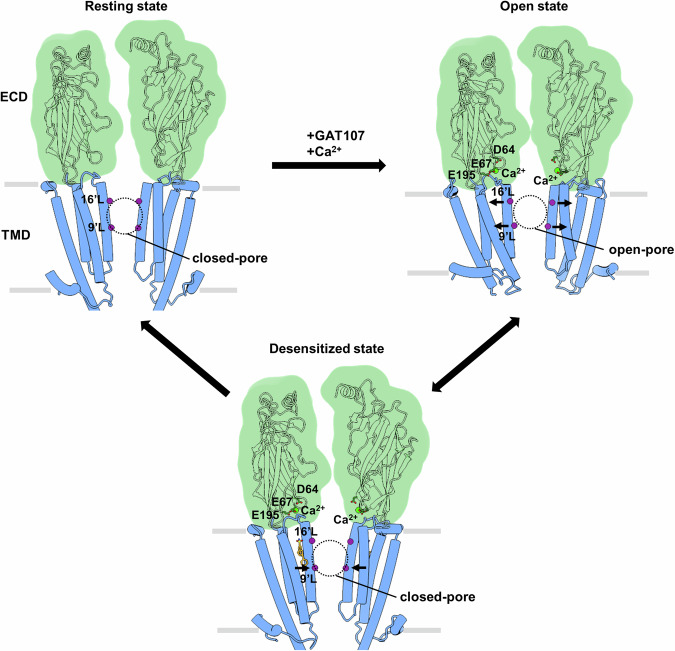
Proposed mechanism of α7 nAChR modulation by ago-PAM GAT and Ca^2+^. In the resting state, the channel pore is closed, and the constriction sites are at 9’L and 16’L. Only two opposite subunits are shown for clarity. In the presence of both GAT and Ca^2+^, calcium ions interact with the acidic residues D64, E67, and E195 in the ECD–TMD coupling region. GAT interacts with the TMDs of the receptor in a highly dynamic mode and leads to the expansion of the channel pore. The transition from the open to desensitized state is coupled with the contraction of the pore, especially in the lower half of the pore. The GAT molecules are located in the intersubunit TMD and maintain the upper half of the pore in a relatively expanded conformation.

On the basis of the cryo-EM structures and electrophysiological experiment results, we proposed a model of α7 nAChR modulation by GAT and Ca^2+^ (Fig. 6). In the presence of GAT and Ca^2+^, Ca^2+^ interacts with the acidic residues (D64, E67 and E195) in the ECD–TMD coupling region. Ca^2+^ may facilitate the weakening or disruption of the intersubunit interaction between K68 and E195, which is crucial for maintaining the resting state, and therefore promotes conformation transition. GAT interacts with the receptor transmembrane helices in a highly variable manner and leads to the expansion of the channel pore to produce an open state. The transition from the open to the desensitized state is coupled with TMD rearrangements, resulting in a contracted channel pore, especially in the lower half of the pore. GAT molecules are located in the intersubunit TMD and maintain the upper half of the pore in a relatively expanded conformation.

In addition to ago-PAM, a series of α7 nAChR structures in complex with agonist/type I or agonist/type II PAMs have been determined^41,43^. As reported recently^43^, agonist/type I PAM complexes were assigned as desensitized states, and agonist/type II PAM complexes were assigned as “desensitized-intermediate” states. Compared to the reported structures, the GAT-desensitized structure in this study is more similar to the agonist/type I PAM-bound complexes. The plots of the channel pore diameter are similar between the GAT-desensitized structure and the agonist/type I PAM complexes. However, there are distinct differences between the channel pore in GAT-desensitized structure and agonist/type II PAM complexes, especially at 9’L. These observations highlight the complexities of α7 nAChR modulation by different types of PAMs. Ago-PAMs of α7 nAChR, such as GAT, stimulate receptor activation via an allosteric site that is distinct from the orthosteric site and enhance orthosteric cholinergic signaling in a positive manner. The dual modes of action exhibited by GAT are expected to increase efficacy and may help overcome the challenges encountered with agonists and PAMs in clinical trials^49^.

Importantly, the anti-inflammatory effect of α7 nAChR activation by GAT was also explored. The diminished IL-1β release from LPS-stimulated microglia indicates the potential role of GAT in treating neuroinflammatory disorders, which is consistent with the findings of α7 nAChR in the cholinergic anti-inflammatory pathway^19^.

Taken together, this study provides insights into the structure and function of human α7 nAChR bound with and modulated by the ago-PAM GAT and Ca^2+^. This study reveals an unconventional allosteric modulation mechanism, which is distinct from typical agonist binding or GAT alone. This work also contributes to a broader comprehension of allosteric modulation in pLGICs.

## Materials and methods

### Construct design and protein expression

To improve expression, the sequence of enhanced GFP was inserted between C412 and S413 in the loop between helices MX and MA of human α7 nAChR (Uniprot: P36544)^41^. Genes were synthesized from Genescript. The DNA encoding the α7 nAChR was cloned into a pcDNA3.1 vector, with a FLAG tag (DYKDDDDK) appended after A502 at the carboxyl terminus, resulting in the construct named α7_EM_ nAChR. For protein expression, HEK293F cells were cultured at 37 °C and 5% CO_2_ in SMM 293-TII expression medium (Sino Biological Inc.) and were used for transfection at a density of 2 × 10^6^ cells/mL. Plasmids for α7_EM_ nAChR and NACHO were co-transfected into HEK293F cells using PEI reagent. Transfected cells were cultured for 60 h before harvesting.

### Protein purification

Cell pellet was resuspended in lysis buffer (150 mM NaCl, 10% Glycerol, 20 mM Tris, pH 8.0). The suspension was supplemented with 1.5% (w/v) *n*-dodecyl-β-d-maltopyranoside (DDM, Anatrace), 0.3% (w/v) cholesteryl hemisuccinate (Sigma) and protease inhibitor cocktail (APExBIO). After incubation at 4 °C for 2 h, the insoluble fraction was removed by ultra-centrifugation at 180,000 × *g* for 45 min at 4 °C and the supernatant was incubated with anti-Flag M2 affinity resin (Sigma) at 4 °C. The resin was then collected and washed with wash (W) buffer (150 mM NaCl, 0.06% GDN, 20 mM Tris, pH 8.0). The proteins were eluted with W buffer supplemented with 200 μg/mL FLAG peptide. After elution, the proteins were concentrated and further purified on a Superose 6 increase column (GE healthcare) equilibrated with 150 mM NaCl, 0.025% GDN, and 20 mM Tris, pH 8.0. Peak fractions of α7 protein were collected and concentrated to ~6 mg/mL. A final concentration of 140 μM GAT107 and 5 mM CaCl_2_ was added into the protein and incubated for an hour before cryo-EM sample verification.

### Cryo-EM sample preparation and data acquisition

A total of 2.5 μL of the sample was applied to freshly plasma-cleaned (H_2_/O_2_, 10 s) holey carbon grids (Quantifoil, R1.2/1.3, 300 mesh, Au). The grids were blotted for 9 s at 100% humidity and 4 °C with a Vitrobot Mark IV (Thermo Fisher Scientific) and plunge-frozen into liquid ethane cooled by liquid nitrogen. The blotted grids were stored in liquid nitrogen until being imaged.

The dataset was collected on a Titan Krios G3i cryo-electron microscope (Thermo Fisher Scientific) operated at 300 kV equipped with a Gatan K3 direct detection camera using the EPU software. Movies were recorded with SA 81,000 × magnification yielding a pixel size of 1.07 Å at University of Science and Technology of China. The total dose of 54 e^–^/Å^2^ was fractionated to 32 frames with 0.125 s per frame. Nominal defocus values ranged from –1.1 to –1.7 μm. A total of 3950 movies were collected.

### Cryo-EM data processing

Dose-fractionated image stacks were subjected to beam-induced motion correction and dose-weighting using UCSF MotionCor2^50^. Contrast transfer function parameters were estimated with Gctf^51^. For particle picking, the representative 2D class averages of apo-α7^41^ were used as templates for auto-picking in relion4.0^52^. Auto-picked particles were extracted in relion and imported into cryoSPARC4 for subsequent 2D classification^53^. Particles from well-defined 2D averages were selected and combined for 3D classification. An ab-initio 3D reconstruction from the 2D average particles was generated in cryoSPARC. The initial model was then used as a reference for 3D classification by heterogenous refinement without the imposition of symmetry. Two selected classes with continuous density for transmembrane helices were used to perform further 3D classification by heterogenous refinement with C5 symmetry imposed in cryoCPARC. Two 3D reconstructions were selected and subjected to further non-uniform refinement, producing the final maps. The overall resolutions were estimated by applying a soft mask excluding detergent micelle and the gold-standard Fourier shell correlation using the 0.143 criterion. Local resolution was determined using cryoCPARC.

### Model building, refinement and validation

Atomic models were built in the software Coot^54^. The model of apo-α7 (PDB: 7EKI) was fitted into the individual EM density maps in chimerax^55^. Every residue was manually examined. The initial model was subjected to iterative manual rebuilding in Coot and real-space refinement in PHENIX^56^. The final model was validated using the module “comprehensive validation (cryo-EM)” in PHENIX^57^. *N*-acetylglucosamine moieties were built to link to Asn46, Asn90 and Asn133 sites, respectively, based on the corresponding densities. For α7/GAT-desensitized structure, the residues from the N-terminal signal peptide (1–22) and the linker between helices MX and MA (349–429) were not built due to the lack of corresponding densities. A cholesterol mimic was assigned according to the extra density nestled between the M3, M4 and MX helices in each subunit. For the α7/GAT-open structure, the residues from the N-terminal signal peptide (1–22), helix MX and the linker between helices MX and MA (345–456) were not resolved due to ambiguous densities.

### Electrophysiology experiments

HEK293T cells were cultured in DMEM medium (Gibco) supplemented with 10% fetal bovine serum (FBS), 100 U/mL penicillin, and 100 U/mL streptomycin at 37 °C in a 5% CO_2_ incubator. 2.5 μg/mL Plasmocin prophylactic (InvivoGen) was added into the medium to prevent mycoplasma contamination. For each transfection in a 24-well-plate well, plasmids encoding α7 nAChR, α7_EM_ nAChR, or α7 nAChR mutants, along with NACHO, were mixed at a mass ratio of 2:1 and diluted in Opti-MEM reduced-serum medium. Subsequently, these plasmids were mixed with lipofectamine 3000 (Invitrogen) prior to being added to the cells. Following a 6-h incubation period, the cells were transferred onto poly-l-lysine-coated slides and cultured for an additional 24–48 h in fresh medium before proceeding to electrophysiological recording.

For whole-cell patch clamp recordings, cells were put in an RC-26 recording chamber (150 μL volume, Warner Instrument) filled with the bath solution containing 150 mM NaCl, 4 mM KCl, 2 mM CaCl_2_, 1 mM MgCl_2_, 10 mM d-glucose, and 10 mM HEPES (pH 7.4). In the calcium-free bath solution, calcium was replaced by 5 mM EGTA. The electrodes were pulled from thick-walled borosilicate glass capillaries with filaments (1.5 mm diameter, Sutter Instruments) on a four-stage puller (P-1000, Sutter Instruments) and had resistances of 2.5–5.5 MΩ when filled with intracellular solution containing 140 mM KCl, 10 mM NaCl, 1 mM MgCl_2_, 5 mM EGTA, and 10 mM HEPES (pH 7.4). All chemicals were obtained from Sigma. Experiments were performed at room temperature with an EPC-10 amplifier (HEKA Electronic) using the data acquisition software PatchMaster. The membrane potential was held at –70 mV in all experiments. Currents were recorded by exchanging the bath solution with the solution containing 1 mM ACh or 10 μM GAT107 with a flow rate of 0.7 mL/min through a 0.7 mm × 0.7 mm square glass tube in the SF-77C fast-step perfusion system (Warner Instrument). Each stimulus of ACh and GAT107 was applied for a duration of 10 s, followed by a 30-s washing-out period. To minimize ligand diffusion and the residual presence of solutions, ensuring the accuracy and reliability of our recordings, all stimuli were separated by at least 5-min intervals of perfusion at a flow rate of 1.0 mL/min. The data were sampled at a frequency of 2 kHz and low-pass filtered at 100 Hz prior to further analysis in Clampfit.

### Calcium flux measurements

HEK293T cells were co-transfected with plasmids encoding α7 nAChR (or its mutants) and NACHO for 24 h and plated into flat-bottom 96-well plates (poly-d-lysine pre-coated) with 100 μL of culture per well (6 × 10^4^ cells per well). Intracellular calcium measurements were performed using fluo-4 (Invitrogen), following the manufacturer’s protocols with a fresh calcium buffer solution (HBSS buffer supplemented with 0.1% BSA, 2.5 mM probenecid, 3 mM CaCl_2_, 1 mM MgCl_2_, pH 7.4). The cells were loaded with 50 μL of the dye per well and incubated at 37 °C for 45–60 min. After a single wash, the cells were further incubated with 40 μL of the calcium buffer. Fluorescence measurements were captured from all wells simultaneously using a FLIPR Penta High-Throughput Cellular Screening System (Molecular Devices). The plates were excited at 488 nm, and fluorescence emission was recorded at 540 nm. Baseline fluorescence was monitored for the initial 20 s, after which 40 μL of GAT107, prepared at twice the final concentration, was added to the cell. Fluorescence intensity was recorded every 1 s for the first 5 min, followed by every 4 s for an additional 8 min. For PNU-120596 measurements, a two-step treatment was applied. Initially, 20 μL of PNU-120596, prepared at four times the final concentration, was added to the cells and incubated for 10 min. Subsequently, 20 μL of nicotine, also prepared at four times the final concentration, was added. The fluorescence intensity was recorded every 1 s for 5 min.

### GAT107 treatment on LPS-simulated BV2 microglial cells

The murine BV2 microglial cell line was purchased from Procell (CL-0493A). Cells were cultured in DMEM medium (Gibco) containing 10% FBS (Gibco) and 1% penicillin-streptomycin (Gibco) in an incubator with 5% CO_2_ at 37 °C.

BV2 cells (5 × 10^5^ cells per well in a 6-well plate) were cultured in MEM medium without FBS for 12 h before adding compounds. Cells were pretreated with GTS21 (100 μM), GAT107 (3 μM or 10 μM), or GAT107 plus MLA (10 μM) for 1 h before being simulated with LPS (1 μg/mL) and incubated for 5 h.

### Quantitative real-time reverse transcription PCR

Total RNA from BV2 cells was isolated using a GeneJET RNA purification kit (Thermo Fisher Scientific). 1 μg of RNA was reverse-transcribed using a Hifair AdvanceFast 1st Strand cDNA Synthesis Kit (Yeasen). 100 ng of cDNA was used as a template for the PCR reaction, which was carried out with Hieff qPCR SYBR Green Master Mix (Yeasen) and primers in a total volume of 20 μL. The amplification was performed through the following steps: 10 min at 95 °C followed by 40 cycles of a two-step loop (20 s at 95 °C and 1 min at 60 °C). The results for target gene expression were normalized to the expression levels of *Actb* gene. The final results were represented as relative values to that of control cells. The primer sets used for PCR were: *Actb*, forward primer 5′-GGCTGTATTCCCCTCCATCG-3′, reverse primer 5′-CCAGTTGGTAACAATGCCATGT-3′; IL-1β, forward primer 5′-GCAACTGTTCCTGAACTCAACT-3′, reverse primer 5′-ATCTTTTGGGGTCCGTCAACT-3′.

### MD simulations

The cryo-EM structures of α7/GAT complex in open and desensitized states were used as the initial models for simulations. For the α7/GAT-open complex, GAT molecules were docked into the TMDs of the receptor using AutoDock Vina. These two GAT-bound complex systems were constructed using Charmm-GUI and employed the Charmm36m force field^58,59^. Counterions (Na^+^ and Cl^–^) were incorporated to neutralize the net system charge, prior to solvation in a TIP3P water^60^ box with a ≥ 10 Å buffer from the protein surface. Preceding production simulations, the system underwent energy minimization utilizing the steepest descent and conjugate gradient algorithms (5000 cycles)^61,62^, followed by gradual temperature annealing from 10 K to 300 K (0.05 ns) under weak harmonic restraints (15 kcal/mol/Å^2^). Subsequently, density and pressure equilibration were conducted for 1 ns under isothermal-isobaric conditions (300 K, 1 atm) employing Langevin dynamics^63^ and Berendsen/Parrinello-Rahman barostats^64,65^. After verifying adequate equilibration, three parallel 500-ns production runs were initiated using GROMACS (version 2022.4)^66^.

## Supplementary information

Supplementary Information

## Data Availability

The cryo-EM maps and the corresponding atomic coordinates of GAT-open and GAT-desensitized states have been deposited in the Electron Microscopy Data Bank (EMDB) and the Protein Data Bank (PDB) under accession codes EMDB-60606 and 9IIV (open), and EMDB-60604 and 9IIR (desensitized), respectively.
