# Supplementary material for: Structural basis for allosteric agonism of human α7 nicotinic acetylcholine receptors
Source: Cell Discov. 2025 Apr 8;11:35. doi: 10.1038/s41421-025-00788-y (PMC11977206; doi:10.1038/s41421-025-00788-y)
Supplement: Supplementary file 1 — Supplementary Information [file 41421_2025_788_MOESM1_ESM.pdf]

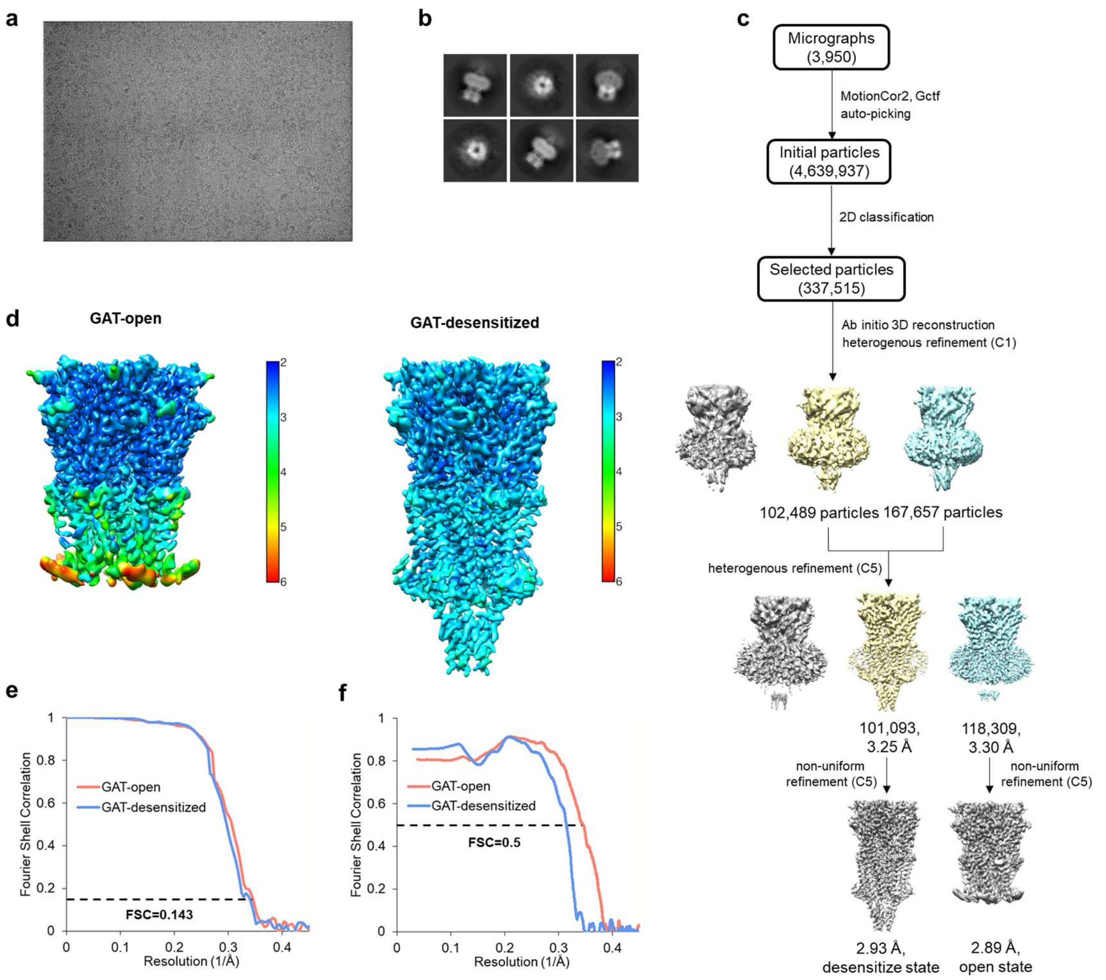

Supplementary Fig. S1 Cryo-EM structure determination of the α7 nAChR in complex with GAT107 and Ca^{2+}. a A representative cryo-EM micrograph. **b** Representative 2D class averages. **c** Cryo-EM data processing flow chart. **d** EM density of two α7 nAChR reconstructions colored according to local resolution estimate. **e** Gold-standard Fourier shell correlation curves from cryoSPARC. **f** Map-to-model Fourier shell correlation curves between each refined model and the corresponding maps.

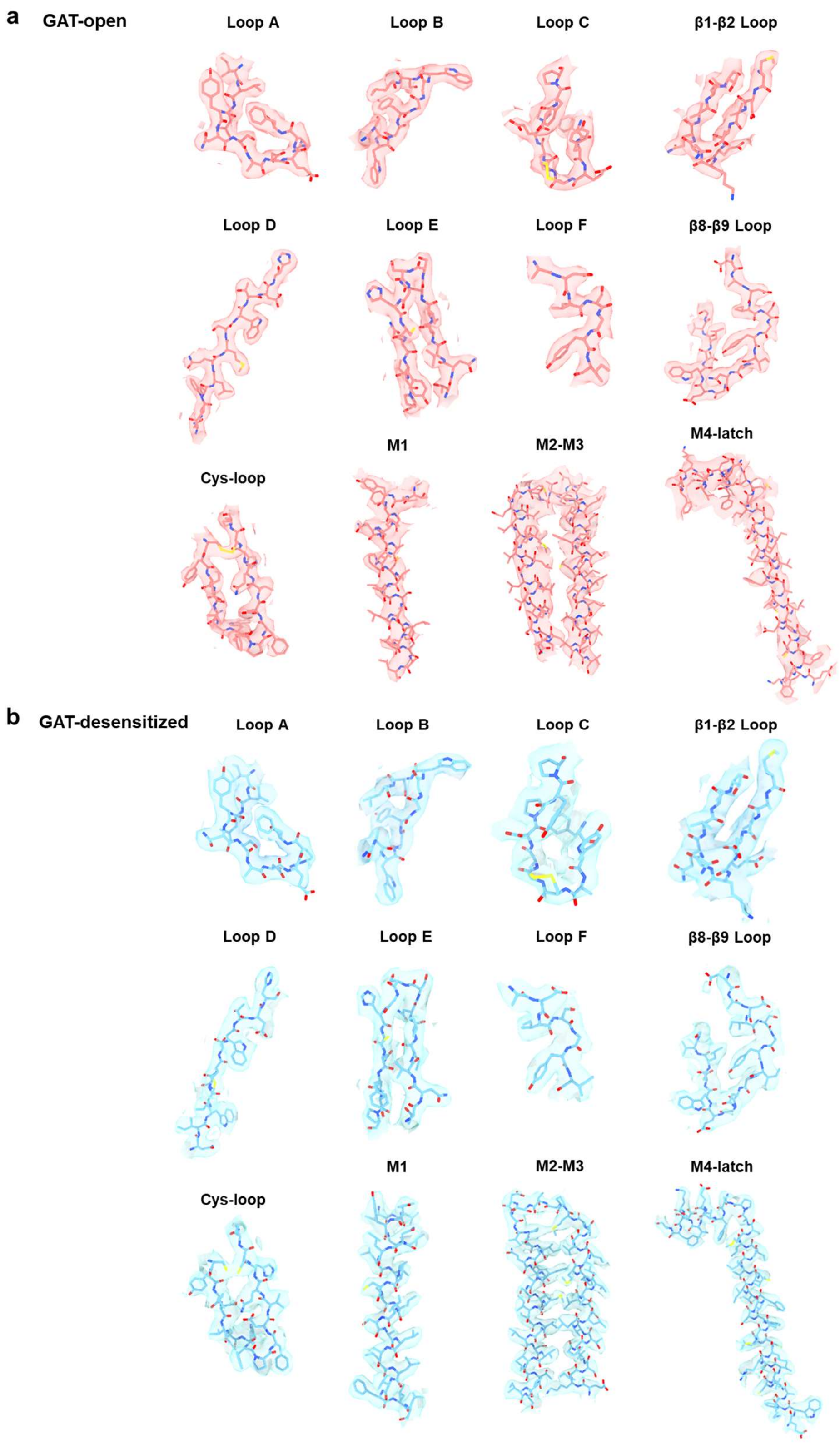

Supplementary Fig. S2 Representative cryo-EM map quality. a Representative densities and fitted atomic models of GAT-open state. Loops A, B, C, D, E, F, β1-β2, β8-β9, and Cys-loop in the ECD, and α-helices M1, M2-M3, and M4-latch in the TMD are shown. **b** Representative densities and fitted atomic models of GAT-desensitized state.

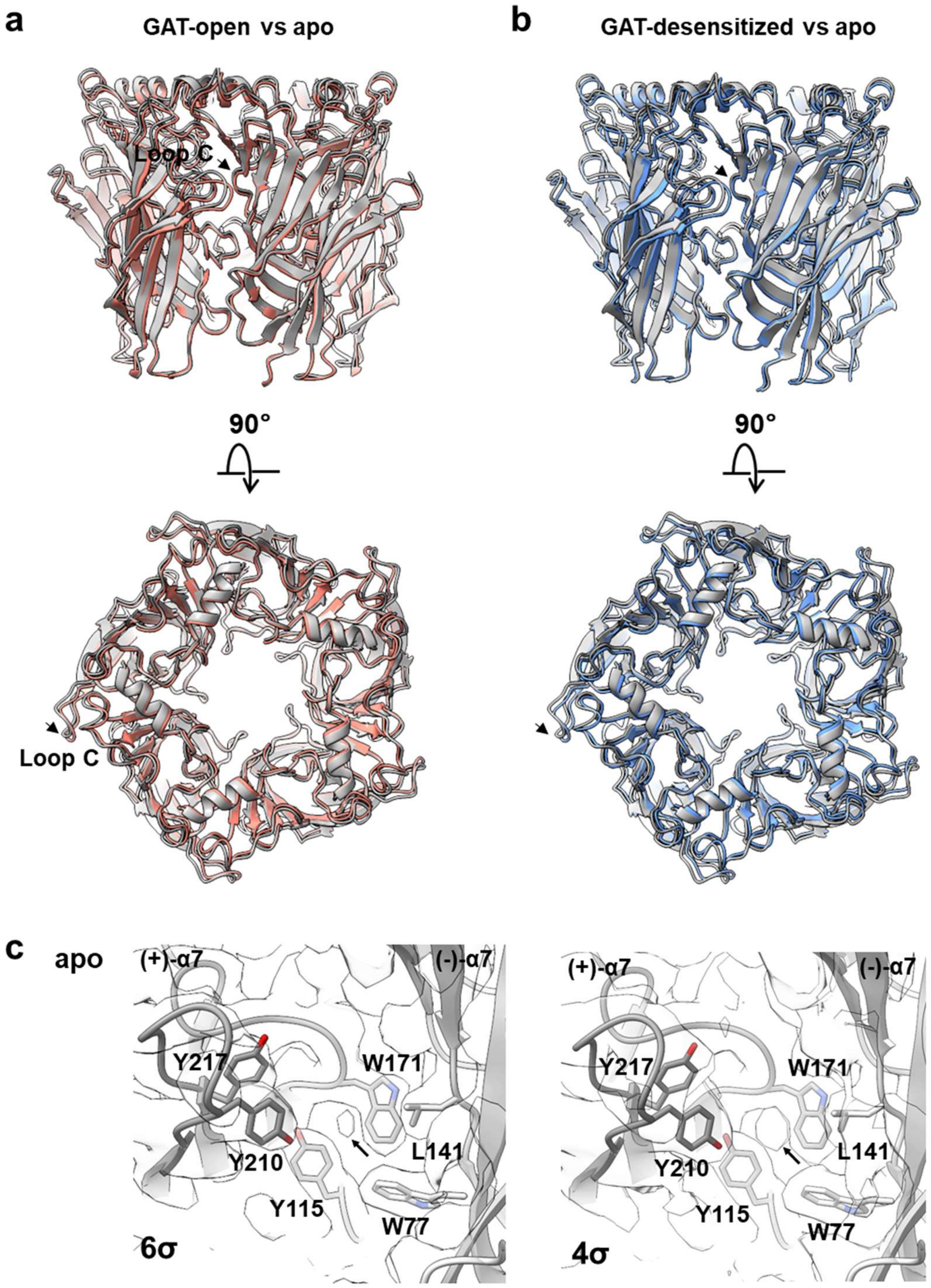

Supplementary Fig. S3 Extracellular domains of α7 nAChR. a Superimposition on the ECDs of GAT-open (orange) and the apo-α7 (grey, PDB: 7EKI) structure. **b** Superimposition on the ECDs of GAT-desensitized (blue) and the apo-α7 (grey) structure. **c** Densities are contoured at 6σ or 4σ in the map of apo-α7. Residues involved in neurotransmitter binding are shown in sticks. The extra density in the orthosteric binding pocket is indicated by the arrow.

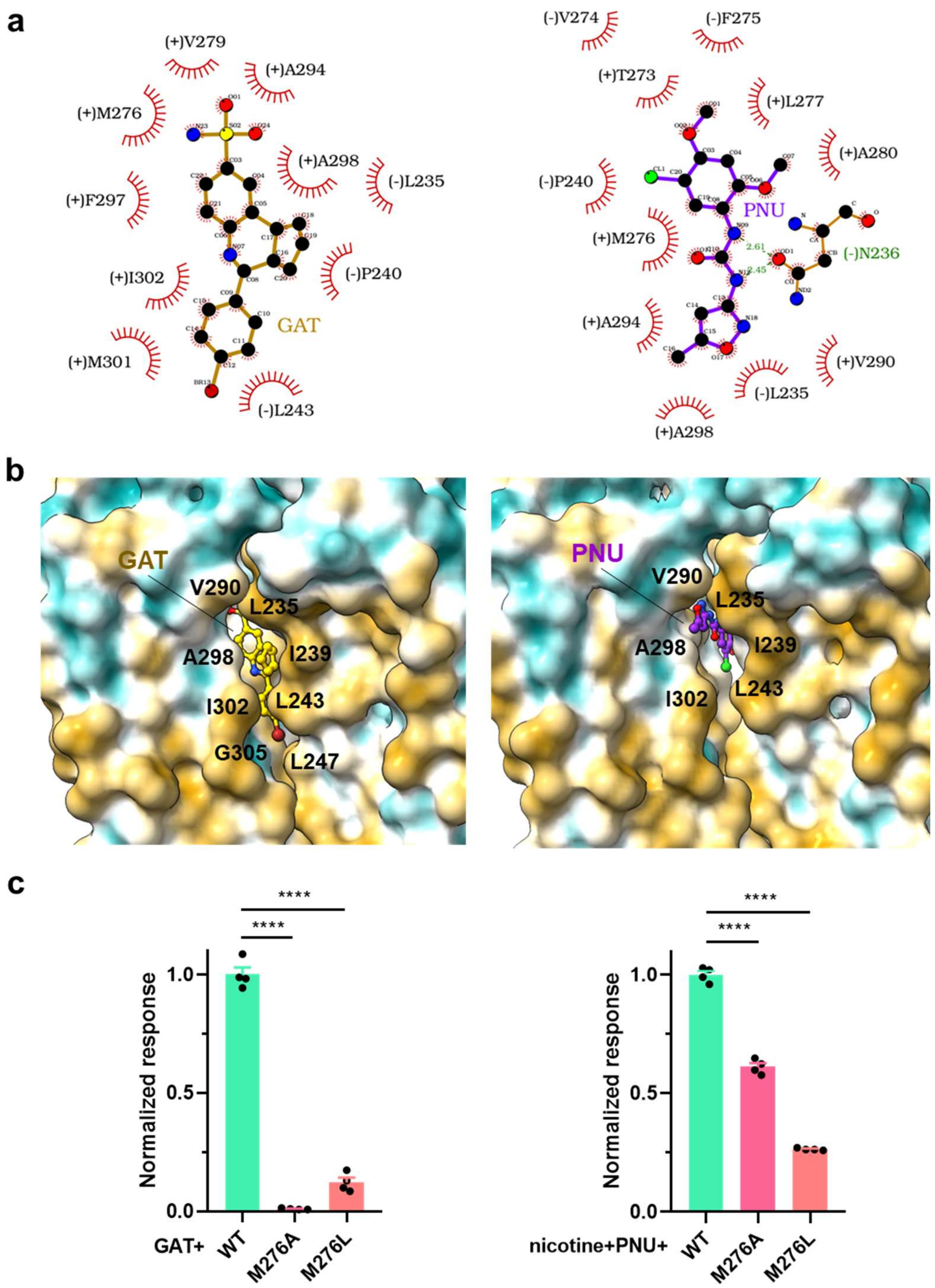

Supplementary Fig. S4 Comparison of the binding sites for GAT and PNU. a Residues that interact with GAT or PNU in the TMD are displayed using LigPlot. **b** The allosteric binding pockets for GAT (left) or PNU (right) are shown as surface with colors ranging from dark cyan (most hydrophilic) to white to dark goldenrod (most lipophilic). **c** The effects of α7 nAChR mutants on GAT or PNU and nicotine were evaluated using calcium flux measurements. The concentrations of GAT, nicotine and PNU used in the experiments are all 10 μM. Data are shown as mean ± SEM ($n \geq 3$). P-values were calculated using an unpaired two-tailed Student's t-test for WT vs. M276L on GAT and WT vs. M276A on PNU (equal variances), and Welch's t-test for WT vs. M276A on GAT and WT vs. M276L on PNU (unequal variances). $^{****}p < 0.0001$.

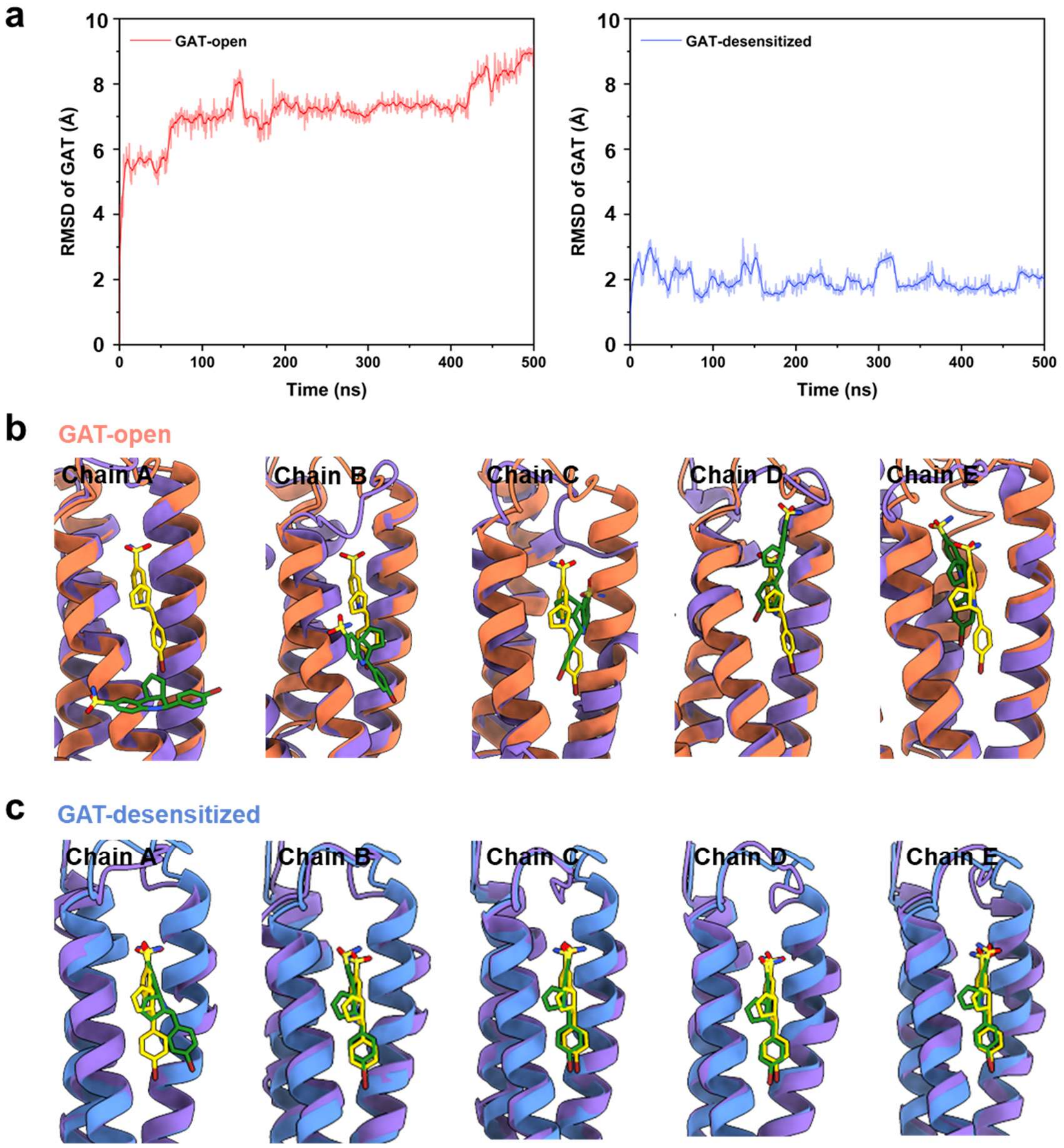

Supplementary Fig. S5 MD simulations of the GAT-open and desensitized states structures. a RMSD values of the GAT molecule in the GAT-open (left) and GAT-desensitized (right) structures. **b** Overlay of the first (orange and yellow) and last (purple and green) frame of a simulation in the GAT-open structure. The TMD in each chain was aligned in the first and last frame. **c** Overlay of the first (blue and yellow) and last (purple and green) frame of a simulation in the GAT-desensitized structure. The TMD in each chain was aligned in the first and last frame.

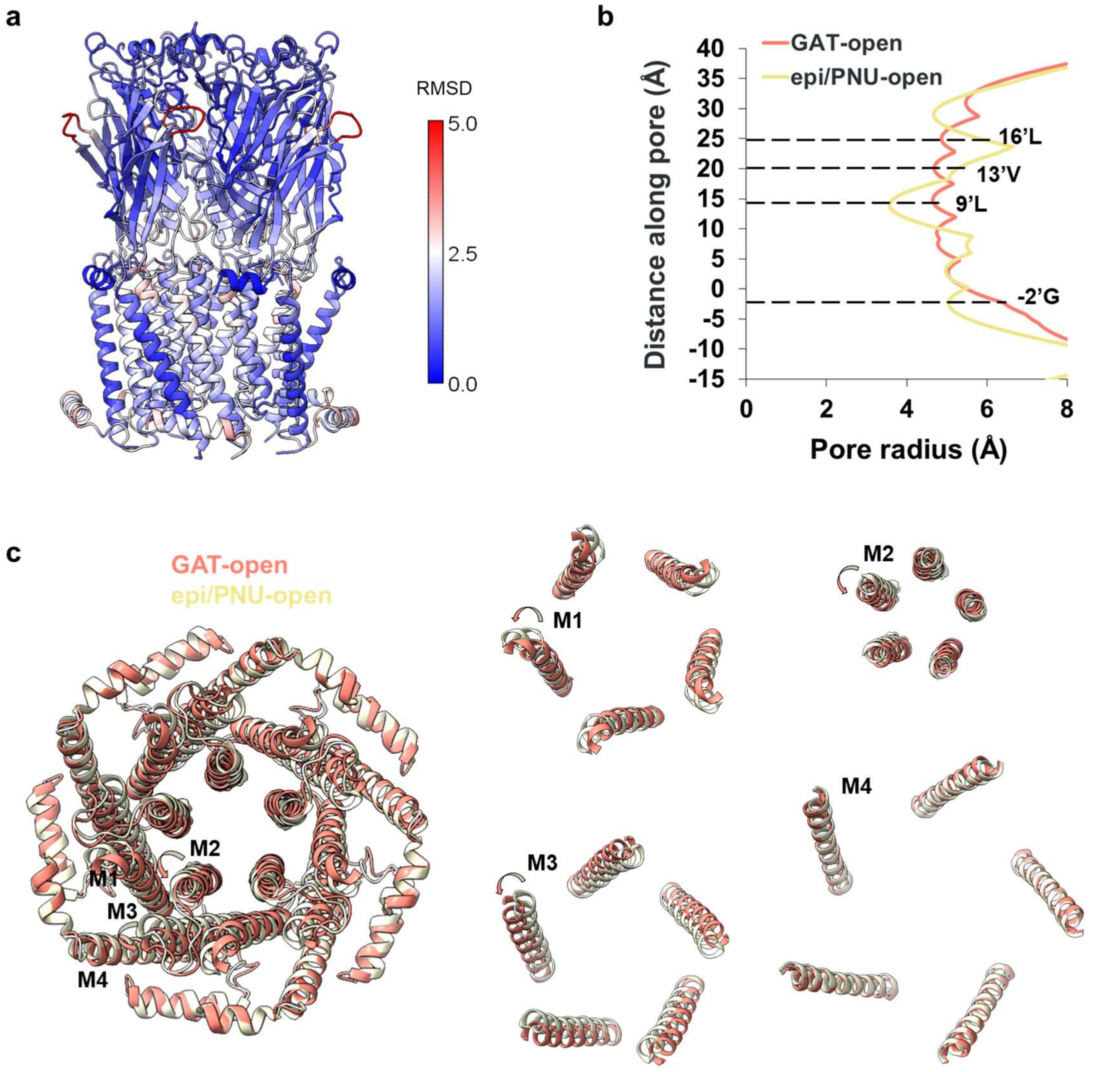

Supplementary Fig. S6 Structural comparisons of GAT-open and epi/PNU-open complex. a The structure differences between the GAT-open and epi/PNU-open states (PDB: 7KOX) are illustrated with the backbone atom RMSD values (0.11 to 10.2 Å). **b** Plots of pore radius for receptors along the pore axis. The α-carbon position of 0' (Lys261) is set to zero. Channel pore radius was calculated using the HOLE program. **c** Comparison of TMDs in GAT-open (orange) with epi/PNU-open (yellow) states.

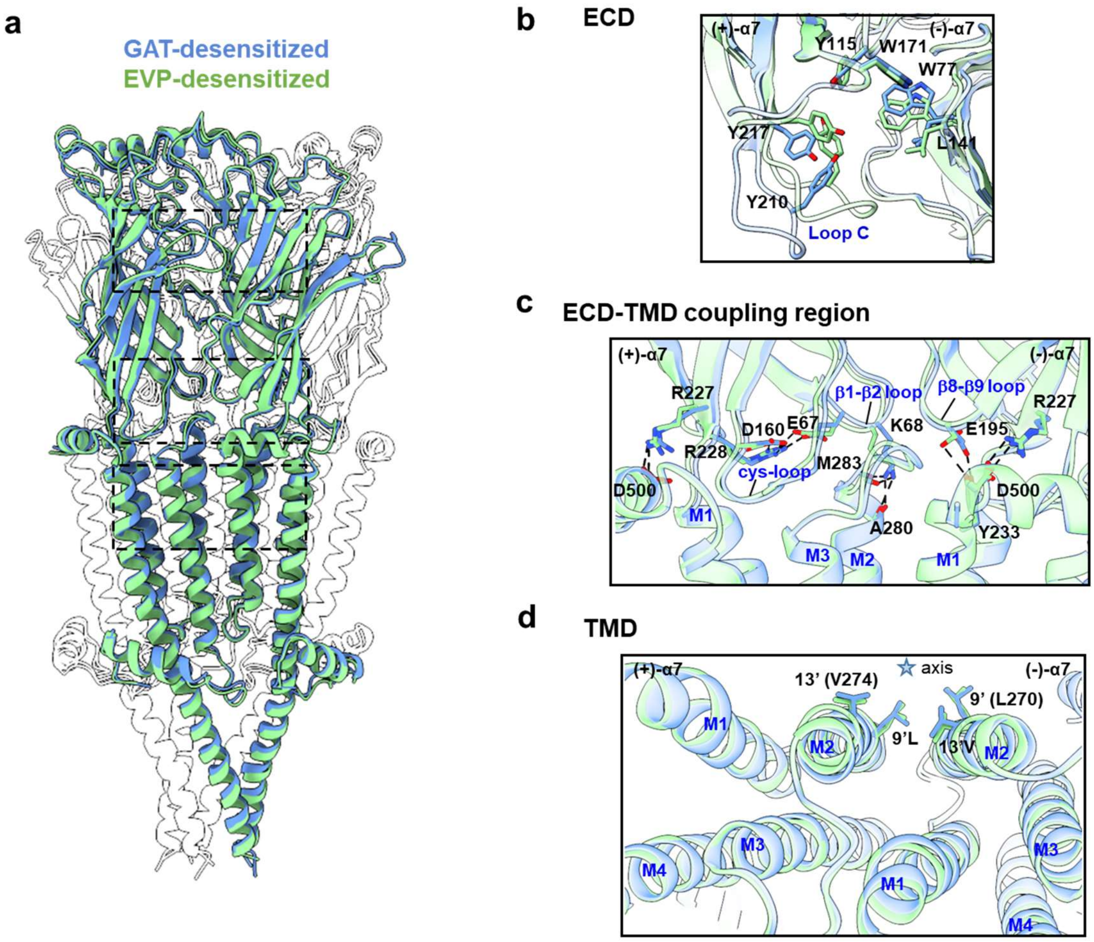

Supplementary Fig. S7 Comparison of the GAT-desensitized state with the α7/EVP structure. **a** Superimposed the GAT-desensitized (blue) and α7/EVP (green, PDB: 7EKP) structures, viewed from the membrane plane. For clarity, all but two of the subunits are mostly transparent. **b** Zoomed view of the ECDs as in (**a**), viewed from the extracellular side. **c** The structural details of the ECD-TMD interface between two adjacent subunits. Electrostatic interactions are represented as dashed lines. **d** The TMDs viewed from the extracellular side.

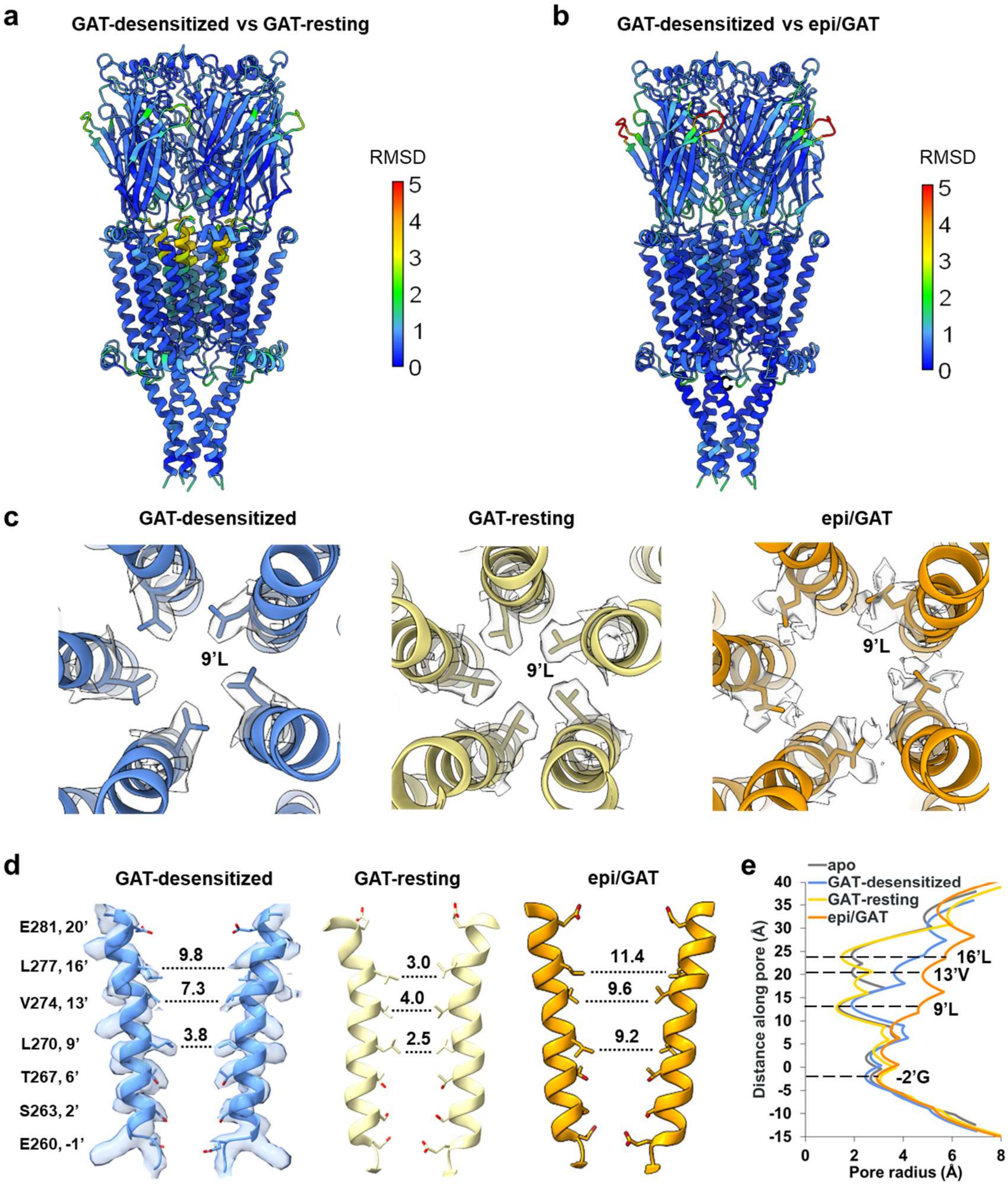

Supplementary Fig. S8 Comparisons of the GAT-desensitized state with the GAT-bound α7 structures published recently. a The differences between GAT-desensitized and GAT resting-like state structure (PDB: 8V86) are illustrated with the backbone atom RMSD values (0.10 to 3.49 Å). **b** The differences between GAT-desensitized and epi/GAT structure (PDB: 8V88) are illustrated with the backbone atom RMSD values (0.02 to 9.58 Å). **c** The 9'L site in the M2 helices is shown with its corresponding density contoured at 4σ, viewed from the extracellular space. **d** Two opposite M2 helices and pore diameters (Å) are shown in each structures. **e** Plots of pore radius for receptors along the pore axis. The α-carbon position of 0'Lys (Lys261) is set to zero. Channel pore radius was calculated using the HOLE program.

Supplementary Table S1 Statistics of cryo-EM data collection, 3D reconstruction, model refinement and validation.

	α7/GAT-open (EMDB-60606) (PDB 9IIV)	α7/GAT-desensitized (EMDB-60604) (PDB 9IIR)
**Data collection and processing**		
Magnification	81,000	81,000
Voltage (kV)	300	300
Electron exposure (e^{-}/Å^2)	54	54
Defocus range (μm)	-1.1~-1.7	-1.1~-1.7
Pixel size (Å)	1.07	1.07
Symmetry imposed	C5	C5
Initial particle images (no.)	4,639,937	4,639,937
Final particle images (no.)	118,309	101,093
Map resolution (Å)	2.89	2.93
FSC threshold	0.143	0.143
**Refinement**		
Model resolution (Å)	2.9	3.2
FSC threshold	0.5	0.5
Map sharpening *B* factor (Å^2)	-126.1	-135.2
Model composition		
Non-hydrogen atoms	14,930	16,430
Protein residues	1835	1990
Ligands	5 (Ca^{2+})<br>20 (NAG)	5 (GAT)<br>5 (Ca^{2+})<br>20 (NAG)<br>5 (cholesterol)
B factors (Å^2)		
Protein	143.16	94.31
Ligand	115.26	122.48
R.m.s. deviations		
Bond lengths (Å)	0.002	0.002
Bond angles (°)	0.480	0.459
Validation		
MolProbity score	1.63	1.47
Clashscore	4.80	5.79
Poor rotamers (%)	1.53	1.14
Ramachandran plot		
Favored (%)	96.42	97.21
Allowed (%)	3.58	2.54
Disallowed (%)	0	0
